# Tannin-Epoxidized Soybean Oil as Bio-Based Resin for Fabrication of Grinding Wheel

**DOI:** 10.3390/polym14245423

**Published:** 2022-12-11

**Authors:** Weicong Wang, Yunxia Zhou, Bowen Liu, Hisham Essawy, Zhiyan Liu, Shuduan Deng, Xiaojian Zhou, Jun Zhang

**Affiliations:** 1 Yunnan Provincial Key Laboratory of Wood Adhesives and Glued Products, Southwest Forestry University, Kunming 650224, China; 2 National Research Centre, Department of Polymers and Pigments, Dokki, Cairo 12622, Egypt; 3 Graduate School of Agriculture, Kyoto University, Kitashirakawa-oiwake-cho, Sakyo-ku, Kyoto 606-8502, Japan

**Keywords:** grinding wheel, tannin, epoxidized soybean oil, hexanediamine, bio-based materials

## Abstract

Formaldehyde-free epoxidized soybean oil-based resin has been prepared under acidic conditions by co-condensation of the epoxidized soybean oil and condensed tannin originating from agricultural and forestry sources as the main raw materials, whereas 1,6-hexanediamine was employed as a cross-linking agent. Fourier transform infrared spectroscopy (FTIR) and electrospray ionization (ESI) corroborated that tannin and epoxidized soybean oil underwent crosslinking under acidic conditions supported by hexamethylenediamine. A bio-based grinding wheel was fabricated by formulation of the developed resin with wood powder as source of grinding particles. The appearance, hardness, compressive strength and wear resistance of the resulting grinding wheel were studied. The results have shown that the grinding wheel possesses a smooth surface with no bubbles or cracks, and its hardness and wear resistance were greater than that of a phenolic resin-based grinding wheel. Interestingly, the grinding wheel incorporates more than 90% of its raw materials as biomass renewable materials; thus, it is generally considered non-toxic. In addition, the future feasibility of this approach to replace some petrochemical resins that are frequently used in the fabrication of grinding wheels is considered.

## 1. Introduction

A grinding wheel is an important abrasive tool in industrial processing. Since the 21st century, phenol-formaldehyde (PF) resin has been widely used as a bonding resin for the preparation of grinding wheels [1,2]. Although it has good strength and heat resistance, free formaldehyde is released during its preparation and use. In addition, phenol, which is its main raw material, comes from the petrochemical industry. Because of global energy shortages and serious environmental pollution, the development of petrochemical materials based on PF resin will be limited in the future. In order to reduce the release of free formaldehyde during industrial production and use, pursue sustainable development and solve the petroleum energy crisis, it is mandatory to develop new trends for the use of biomass materials to partially or completely replace petrochemical materials. The research on biomass renewable materials from agricultural and forestry crops, such as cellulose [3,4], condensed tannin [5,6], starch [7,8] and lignin [9,10], is gradually increasing. Among them, condensed tannin from bark is emerging in the field of adhesives [11,12,13], foam [14,15,16,17,18], plastics [19], building materials [20] and wear-resistant materials [21,22,23]. Specifically, condensed tannin is used to prepare resin-based grinding wheels in place of traditional PF resin because of its high hardness after curing. At first, tannin was used to co-polycondensate with a biomass material such as furfuryl alcohol under acidic conditions for preparation of tannin-furfuryl alcohol resin-based grinding wheels [22,23]. Although the grinding wheel acquired high hardness, it was brittle. It is easy for furfuryl alcohol to undergo self-polycondensation under acidic conditions [19], which makes it not suitable for reactions with tannin. To improve its reactivity, formaldehyde, glyoxal and epoxy resin [13] were employed as cross-linking agents. Although the obtained grinding wheel showed good mechanical properties, the high cost of furfuryl alcohol and intensive release of heat under strong acidic conditions make the process unsafe and not conducive to industrial Application.

Furthermore, as a biomass material, epoxidized soybean oil has a highly active epoxy group. Previous studies have shown that epoxidized soybean oil can react with tannin to form flexible foam [24]. Tannin is degraded and epoxidized before reacting with epoxidized soybean oil. Although the foam prepared has certain strength, its process was cumbersome whereas the yield was low. Therefore, in this study, hexanediamine was used as a crosslinking agent to improve the reactivity of condensed tannin and epoxidized soybean oil, which simplified the preparation process and facilitated preparation of a new type of tannin-epoxidized soybean oil-hexanediamine (TEH) resin-based grinding wheel. Further, a previous work was conducted, in which grinding wheels were formulated with aluminum oxide and silicon dioxide, which are the main grinding materials [5,11]. Nevertheless, the large particle size and loading percent rendered the mixing process difficult and the resulting mixes experienced uneven distribution of the grinding particles in the resin, which limits its applicability. Here, in this study, wood powder, coffee powder and walnut shell powder with smaller particle sizes and loading were used to replace aluminum oxide and silicon dioxide, so that the distribution of grinding particles became more uniform, whereas the biomass content of the grinding wheel was increased to more than 90%. This research provides a research basis for the development of an environmentally friendly biomass-based grinding wheel with low cost and easy preparation, and has certain application value.

## 2. Materials and Methods

### 2.1. Materials

Larch (*Larix gmelinii* (Rupr.) Kuzen.) tannin powder (T) was purchased from Wuming tannin extract factory (Nanning, China); 1,6-hexanediamine (H) was purchased from Hongting industrial company (Zhengzhou, China); Eucalyptus wood powder (*Eucalyptus robusta Smith*), 150 mesh, Rubiaceae (*Rubiaceae Juss.*) coffee powder (*Coffea arabica*), 150 mesh and Walnut (*Juglans regia* L.) shell powder and 150 mesh were purchased from Guangzhou Chaobiao Sawmill (Guangzhou, China); Epoxidized soybean oil (E, 98%) was purchased from Weizhen chemical company. (Jinan, China); P-toluene sulfonic acid (pTSA, 65% aqueous solution), phenol (P, 80%) and formaldehyde (F, 37%) were purchased from Sinopharm company (Beijing, China). 

### 2.2. Preparation of TEH Resin-Based Grinding Wheel

First, 1,6-hexanediamine was heated to 90 °C for liquification, and then 9 mL of epoxidized soybean oil and 30 g of 1,6-hexanediamine were mixed in a 500 mL beaker using a magnetic stirrer (DF-101, Yuhua instrument company, Shanghai, China) for 30 min in a water bath at 90 °C to obtain the epoxidized soybean oil-hexanediamine (EH) resin. Then, 30 g of tannin and 2 g of pTSA were added under stirring over 5 min to obtain TEH resin.

PF resin was also prepared [23] by mixing phenol (80%) and formaldehyde (37% aqueous solution) (F/P = 2.2) in a beaker, in which the formaldehyde was added in quartiles, whereas pTSA was used to adjust the pH value of the solution to 2 throughout the process. Accordingly, after mixing the first portion of formaldehyde with phenol via stirring, the solution was heated to 94 °C. The interval between adding the next amount of formaldehyde was fixed to 15 min. After adding the last formaldehyde portion, the reaction was continued for 1 h then cooled down to obtain the PF resin.

**Scheme 1:**
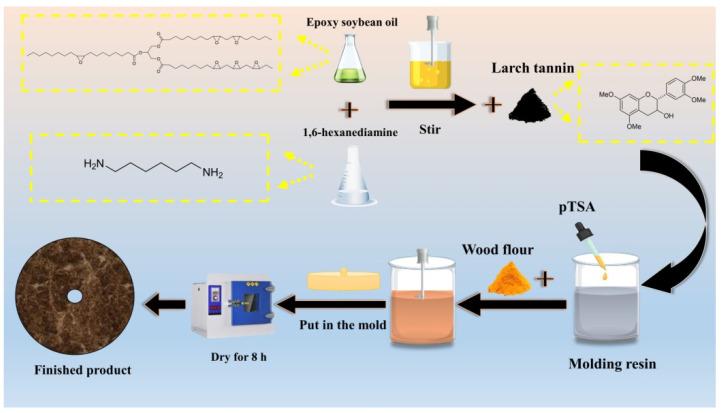
Preparation process of S4.

Secondly, the TEH resin was mixed with wood powder, coffee powder and walnut shell powder, individually, (the powder accounts for 30% with respect to the TEH resin) under stirring for 5 min. Then, the mixtures were put individually into a mold with an outer diameter of 120 mm, inner diameter of 6 mm and thickness of 6 mm. The molds were then transferred to an oven (GZX-GF101-3BS-II, Shanghai Yuejin Medical Equipment Co., Ltd., Shanghai, China) set at 100 °C for 12 h. The obtained grinding wheels are termed as follows: S1 (no powder), S2 (with coffee powder), S3 (with walnut shell powder) and S4 (with wood flour). Under the same experimental conditions, a grinding wheel was prepared based on PF resin with 30% wood powder (PFG). Table 1 shows the formulations of the various grinding wheels. The preparation process of S4 is shown in Scheme 1.

### 2.3. Characterizations

The prepared grinding wheel samples were placed in a room at 20 °C and 50% relative humidity for 24 h. Subsequently, the samples were subject to different evaluation tests and the average of five measurements was considered.

The T, EH and TEH resins were examined with infrared spectrometer Varian 1000 (Varian, Palo Alto, CA, USA). Thus, one gram of KBr was mixed with 0.01 g of each cured resin after crushing into fine powder and the test samples were scanned over a wavenumber range of 500 to 4000 cm^−1^. Meanwhile, Waters Xevo triple quadrupole-mass spectrometer (Waters, Milford, MA, USA) equipped with an electrospray ionization source (ESI) was used for elucidation of the chemical structure of the TEH resin. The TEH resin was successively dissolved in chloroform with a concentration of 10 μg/mL, and injected into the ESI source plus ion trap mass spectrometer (Bruker Daltonics Inc., Billerica, MA, USA) through a syringe at a flow rate of 5 μL/s. The spectra were recorded in a positive mode with ion energy of 0.3 eV and a scanning range of 0 Da to 2000 Da.

The grinding wheels were cut into a size of 10 mm × 10 mm × 10 mm for observation of their morphology using a scanning electron microscope (SEM, S4800, Hitachi, Tokyo, Japan).

The thermal degradation behavior was investigated using a thermogravimetric analyzer (TGA 209 F3, Netzsch, Selb, Germany) under a nitrogen atmosphere at a heating rate of 20 °C/min. from 30–900 °C.

Differential scanning calorimeter (DSC, 204 F1, Netzsch, Selb, Germany) was used to study the curing behavior of the resin under nitrogen atmosphere at a heating rate of 10 °C/min in the temperature range of 30–250 °C.

Brinell hardness tester (HB-3000B, Sidewall Photoelectric Instrument Factory, Beijing, China) was used to measure the Brinell hardness (N·mm^−2^) of the grinding wheels, referring to the national standard GB/T231.2-2012. A hard alloy ball with a diameter of 10 mm was used to test samples with a size 50 mm × 50 mm × 25 mm under a maximum force of 2452 N for 60 s. The Brinell hardness was calculated according to Equation (1).

(1) $$\mathrm{Brinell}\;\mathrm{hardness}=0.102\frac{2F}{\pi D\left(D-\sqrt{D^{2}-d^{2}}\right)}$$

where 0.102 is a constant; *F* is the maximum force, N; *D* is the diameter of the hard alloy ball, mm and *d* is the average diameter of the indentation, mm.

Samples of 25 × 20 × 15 mm size were used for testing the compressive strength according to the national standard GB/T14208.1-2009 using a universal mechanical testing machine (INSTRON-4467, Boston, MA, USA), which was operated at a loading rate of 2.0 mm/min.

A Taber abrader (ZOT-6013TABER, Sivaca, Guangzhou, China) was used to evaluate the wear resistance of the grinding wheel. H18 grinding wheels, fixed on the machine, were used for testing at 60 rpm for a total of 500 cycles. The wear index was obtained from Equation (2):

(2) $$\mathrm{wear}\;\mathrm{index}=\frac{W_{1}-W_{2}}{N}\times 1000$$

where *W*_1_ is the weight of the grinding wheel before the test; *W*_2_ is the weight of the grinding wheel after the test and *N* is the number of cycles.

## 3. Results and Discussion

**Figure 1:**
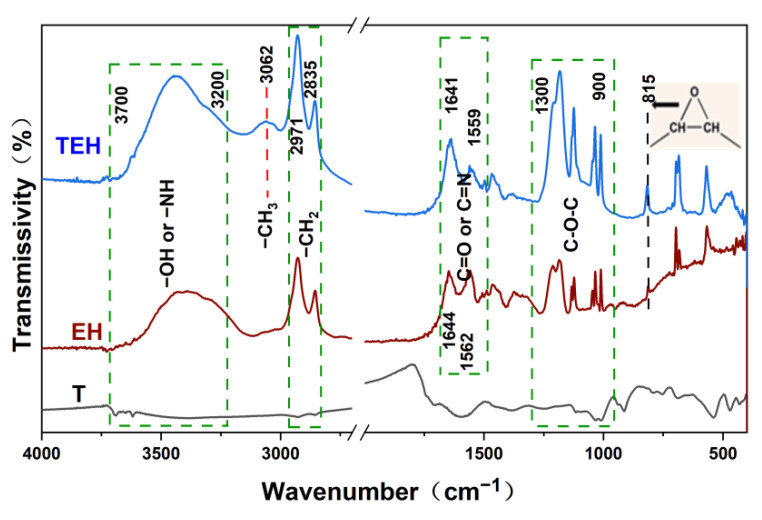
FT-IR spectra of tannin (T), epoxidized soybean oil-hexanediamine (EH) and tannin-epoxidized soybean oil-hexanediamine (TEH) resins.

Figure 1 shows the FT-IR spectra of T, EH and TEH resins. It can be seen that the –OH or N–H stretching vibration appears at 3700–3200 cm^−1^, which is caused by the epoxy ring opening of the epoxy group in epoxy soybean oil and the amino group at the end of hexanediamine, and the hydroxyl generated by ring opening in the product. However, the amino group still exists at the end of the polymer generated in the system, and some amino groups can form strong hydrogen bonds with adjacent hydroxyl groups. The peak at 3062 cm^−1^ reveals the stretching vibration of –CH_3_, which shows a strong absorption in the case of TEH, indicating that the ring opening reaction of the epoxy ring from soybean oil produces more –CH_3_. The absorption peaks at 2971 and 2835 cm^−1^ are ascribed to symmetric and anti-symmetric stretching vibrations of methylene groups, respectively, whereas the peak at 1644–1559 cm^−1^ refers to the stretching vibration of C=O of epoxidized soybean oil ester or C=N produced from the reaction with hexanediamine. In this range, the presence of tannin as part of TEH resin introduces a long range of conjugation comprising the benzene ring, which homogenizes the electrons of reactants, thus causing the absorption peak of C=O or C=N in the TEH spectrum to undergo a shift compared with that of EH. Further, the peak at 1300–900 cm^−1^ indicates the symmetric stretching vibration of C–O–C, giving rise to the ring opening reaction of the epoxy ring of soybean oil by hexanediamine, and an electrophilic reaction took place with tannin, leading to the formation of a network structure.

**Figure 2:**
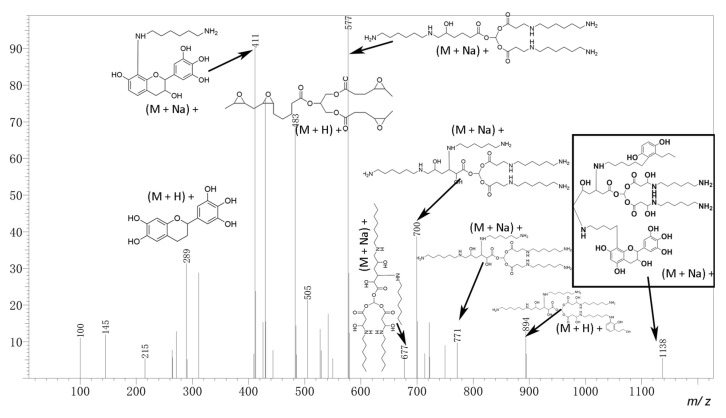
ESI-MS spectra of the tannin-epoxidized soybean oil-hexanediamine (TEH) resin.

**Scheme 2:**
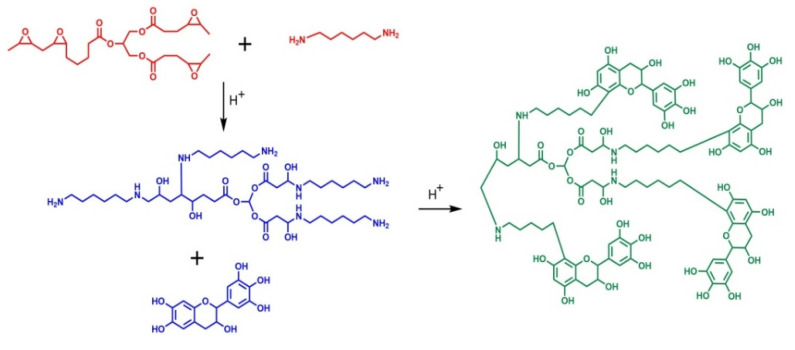
Main reaction processes of epoxidized soybean oil with hexanediamine and tannin under acidic conditions.

Figure 2 shows the ESI-MS spectrum of TEH resin. The 289 Da and 483 Da bands peaks are attributed to the structure of the tannin monomer and epoxidized soybean oil respectively, indicating that some of the tannin and epoxidized soybean oil did not participate in the reaction. At the same time, the 411 Da absorption peak is attributed to the reaction of tannin with hexanediamine. On the other hand, the 577 Da, 677 Da, 700 Da and 771 Da bands are attributed to the ring opening reaction of the epoxy group of epoxidized soybean oil with hexanediamine, and more importantly, the 894 Da and 1138 Da absorption peaks are attributed to higher oligomeric species derived from the co-reaction of tannin, epoxidized soybean oil and hexanediamine. Based on the results of ESI-MS and FT-IR, it is supposed that under acidic conditions, the epoxy group of epoxidized soybean oil reacts with the amino group at one end of the hexanediamine to form a ring opening adduct, whereas the tannin is activated under the same conditions so that the C6 and C8 positions in the A-ring of tannin shows high reactivity [25,26,27]; therefore, the amino group at the other end of the hexanediamine reacts with the C6 or C8 sites of A ring in activated tannin to form a stable network structure (Scheme 2).

**Figure 3:**
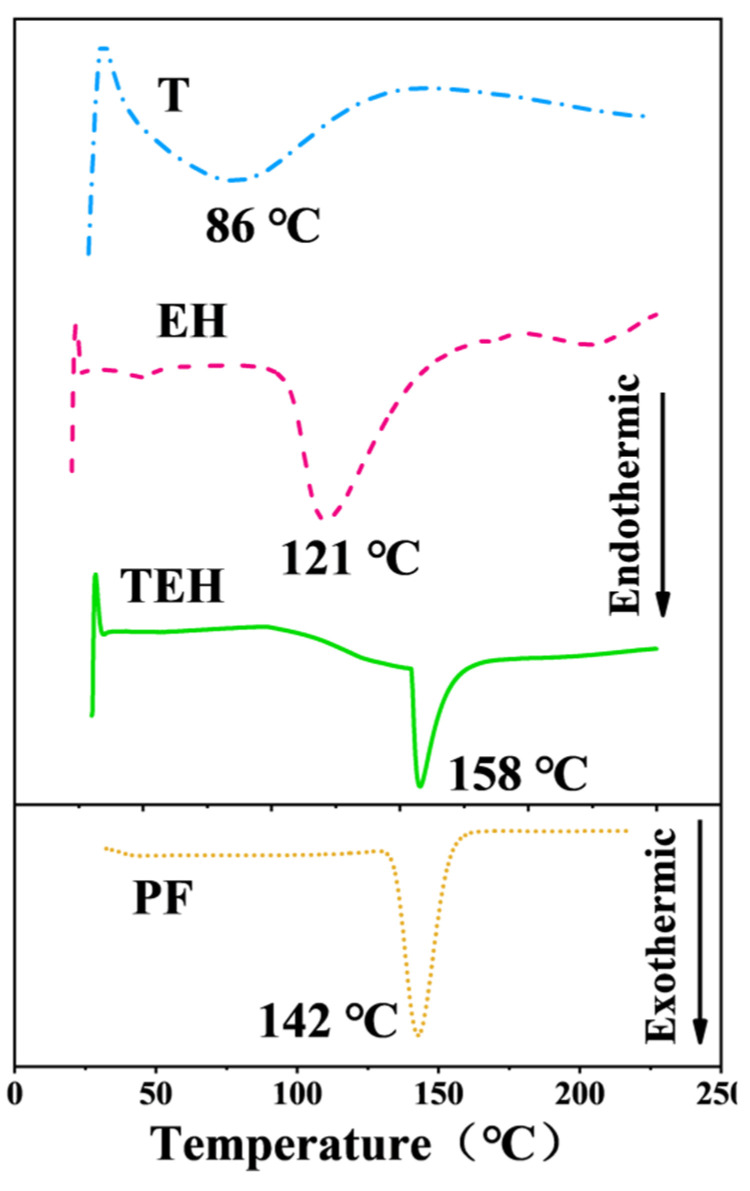
DSC thermograms of the tannin (T), epoxy soybean oil-hexanediamine (EH) and tannin-epoxidized soybean oil-hexanediamine (TEH).

Figure 3 displays the DSC thermograms of T, EH, TEH and PF resins. T, EH and TEH resins show an endothermic peak, whereas on the contrary, PF resin shows an exothermic peak. The polycondensation curing temperature of T is about 86 °C, whereas the ring opening reaction of epoxidized soybean oil and hexanediamine is mainly about 121 °C. It is worth noting that after adding tannin, the main polycondensation reaction temperature rises from 121 °C to 158 °C. According to the FT-IR and ESI-MS results, under acidic conditions, the amino group of hexanediamine can react with the C6 or C8 positions of the tannin of A ring, making the EH system more complex. At the same time, the curing temperature of TEH resin is close to PF resin (142 °C), indicating that it is feasible to use TEH resin to replace PF resin in industrial production.

**Figure 4:**
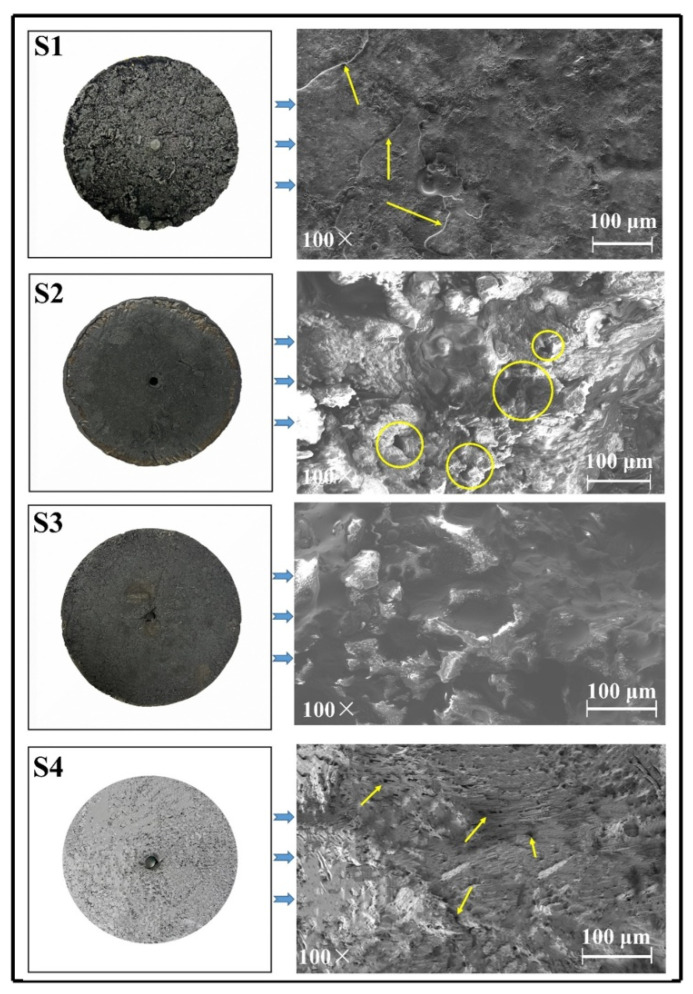
SEM micrographs of the various grinding wheels.

Figure 4 shows the visual appearance and SEM images of the different grinding wheels. It can be seen from the appearance of the four groups of grinding wheels that although all grinding wheels are formed after curing, the surface of the grinding wheel S1 is relatively rough. On the contrary, the surface of the grinding wheel with coffee powder (S2), walnut shell powder (S3) and wood powder (S4) is relatively smooth. No bubbles were detected on the surface of sample S1, whereas a high brittleness related to that tannin has a greater phenolic ring structure [19]. The addition of tannin increases the brittleness of the EH system, making the S1 material surface to exhibit cracks (shown by the arrow). On the contrary, increasing the biomass powder can improve the toughness of the grinding wheel and reduce cracks. However, there are many pores that can be recognized on the surface of sample S2 (marked by a circle), which is attributed to the light weight of the coffee powder and its known tendency to agglomerate in water [28,29], so its compatibility with TEH resin system was poor. Compared with S2 and S3, there were no pores or cracks on the surface of the S1 sample; however, the walnut shell powder-based wheel was hard [30,31], whereas the powder shape was not uniform and the distribution in the TEH system was uneven, where some portions of the resins were not filled with the walnut shell powder. In the case of sample S4, although there were tiny pores (indicated by arrow), the wood powder was evenly dispersed in the TEH resin.

**Figure 5:**
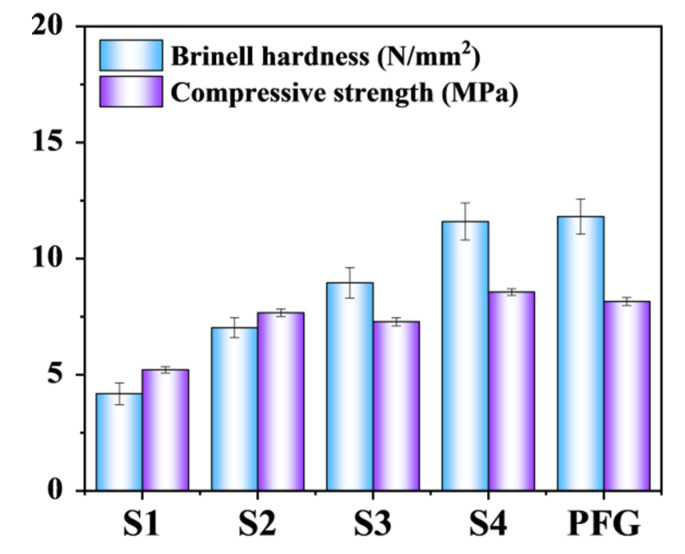
Brinell hardness and compressive strength values of the different grinding wheels.

Figure 5 shows the Brinell hardness and compressive strength of the prepared grinding wheels. From the hardness results, compared with sample S1 (4.18 N/mm^2^), the addition of biomass powder into TEH resin has greatly improved the Brinell hardness of the grinding wheels, which reached 7.02 N/mm^2^, 8.96 N/mm^2^ and 11.60 N/mm^2^, for S2, S3 and S4, respectively. The Brinell hardness of S4 was the highest, which was correlated from the SEM images in the sense that the compatibility of wood powder in THE resin was better than either the coffee or walnut shell powders, which caused the greater hardness. More importantly, the Brinell hardness of S4 is close to that of the PF resin-based grinding wheel (PFG) (11.81 N/mm^2^). At the same time, the compressive strength results are consistent with the results of Brinell hardness. The compressive strength of sample S4 (8.56 MPa) is higher than that of S1, S2 and S3 samples. Moreover, the compressive strength of S4 is even higher than that of PFG (8.16 MPa).

**Figure 6:**
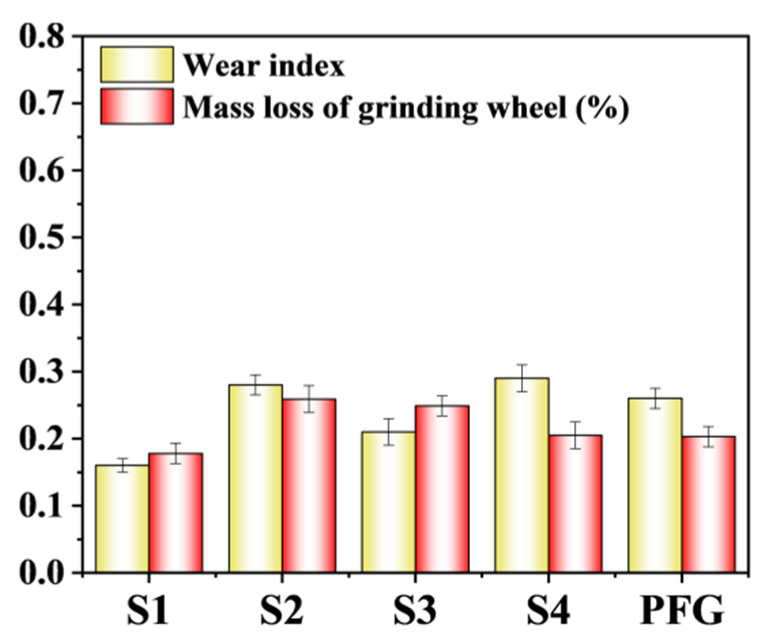
Abrasion resistance of the different grinding wheels.

Figure 6 shows the wear resistance data of samples S1, S2, S3, S4 and PFG. After the H18 grinding wheel was ground 500 times at 60 rpm, the mass loss of sample S1 was 0.178%, and the wear index was 0.16. Compared with sample S1, the mass loss of samples S2, S3 and S4 reached 0.259%, 0.249% and 0.205%, respectively. This shows that the addition of biomass powder caused destruction of the molecular structure of resin during the blending between the powder and resin [2,5], which reduces the stability and strength of cured TEH resin. Among the S2, S3 and S4 grinding wheels, the mass loss of sample S4 was the lowest. Interestingly, the addition of biomass powder caused the wear indexes of S2, S3 and S4 grinding wheels to increase to 0.28, 0.21 and 0.29, respectively. These results show that sample S4, which is made of wood powder, acquired a higher wear index and lower mass loss compared with S2 and S3, which were made of coffee and walnut shell powder, respectively. Moreover, the wear index of S4 was even higher than that of PFG (0.26), and both of them have similar mass loss. Therefore, the test results of compressive strength and Brinell hardness testify that the grinding wheel made of TEH resin is competitive with that of PF resin.

**Figure 7:**
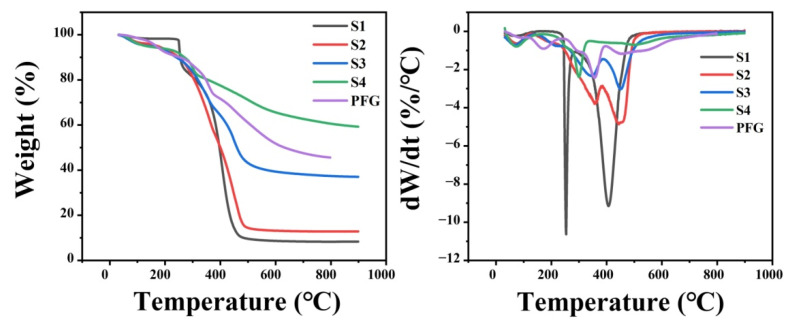
TGA and DTG curves of the different grinding wheels.

Figure 7 shows the profile of the thermal degradation behavior of the different grinding wheels. The relevant TG and DTG curves obviously show that between 30 °C and 200 °C, the mass loss and thermal degradation rate of samples S2, S3, S4 and PFG were higher than that of sample S1, which depends on the fact that the mixing of biomass powder and resin destroys the structure of the resin matrix, causing small molecules to volatilize at higher temperatures. Moreover, the increase in temperature between 200 °C and 500 °C was met by the appearance of two high peaks in the DTG thermogram, which present breakage between molecular chains and volatilization of more biomass powder [2]. Among them, the mass loss of sample S1 was the largest, which indicates that adding biomass powder can improve the thermal stability of the grinding wheels at high temperatures, leaving mass loss in the case of S4, which was lower with respect to S2 and S3. This shows that the thermal stability of wood powder is greater compared with coffee powder and walnut shell powder. Furthermore, the lower mass loss of S4 compared to PFG shows that the thermal stability of TEH resin is larger than that of PF resin, which depends to some extent on the structure of the polyphenol ring in tannin [19].

## 4. Conclusions

Soybean oil can be easily epoxidized and reacted with condensed tannin from agricultural and forestry sources via co-condensation in the presence of 1,6-hexanediamine as a crosslinker under acidic conditions to yield formaldehyde-free resin with great potential to replace resins derived from the petrochemical industry for application in many fields. Among which, the developed resin can be utilized for fabrication of grinding wheels when combined with wood powder as a source of grinding particles. The proceeding crosslinking reactions can be simply proven via techniques such as FTIR and ESI, which supported the promotion of crosslinking in the presence of hexamethylenediamine. The produced grinding wheels possessed good appearance and a smooth surface lacking any bubbles or cracks, as was indicated by SEM, whereas mechanical parameters such as hardness, compressive strength and wear resistance were greater than those of phenolic resin-based grinding wheels. The grinding wheels prepared by this approach are very interesting compared with their counterparts fabricated from phenolic resin, considering that 90% of their incorporated raw materials are biomass renewable components and are thus, addressing health concerns, non-toxic. From an environmental concern perspective, their future feasibility as substitutes for certain petrochemical resins, which are regularly used in the fabrication of grinding wheels, will be highly appreciated.

## Figures and Tables

**Table 1:** Formulations of the various grinding wheels.

Sample	Type of Resin	Type of Abrasive Particles	Resin (g)	Abrasive Particles (g)
S1	TEH	/	60	/
S2	TEH	coffee powder	60	18
S3	TEH	walnut shell powder	60	18
S4	TEH	wood flour	60	18
PFG	PF	wood flour	60	18

## Data Availability

Not applicable.
